# Supplementary figures and images for: Researchers collaborate with same-gendered colleagues more often than expected across the life sciences
Source: PLoS One. 2019 Apr 26;14(4):e0216128. doi: 10.1371/journal.pone.0216128 (PMC6485756; doi:10.1371/journal.pone.0216128)

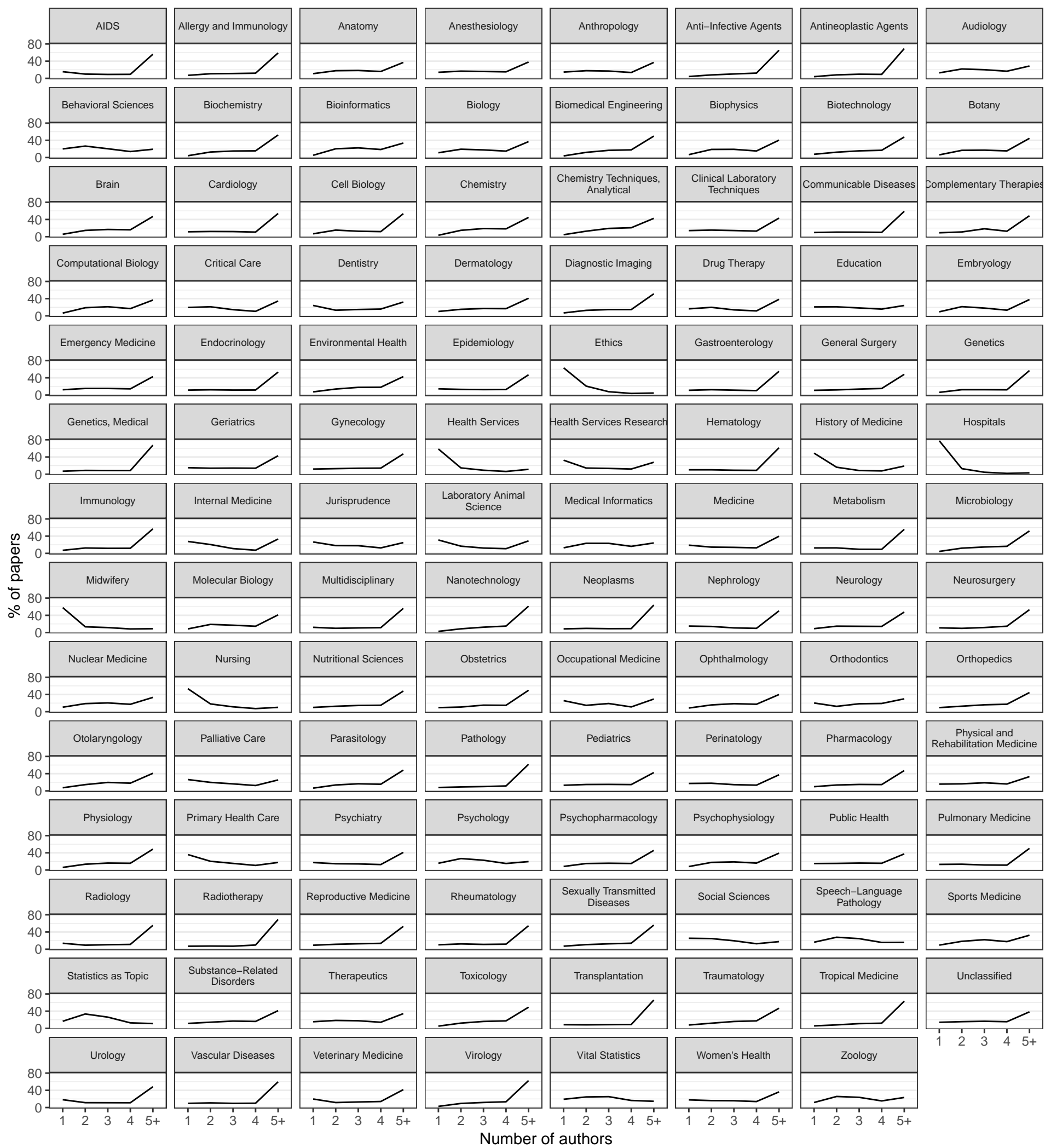

Supplement: S1 Fig — This information can also be found in S3 Data. (PDF) [file pone.0216128.s001.pdf]

Number of journals

60

40

20

0

-0.2

0.0

0.2

Change in  $\alpha'$  over the last ten years

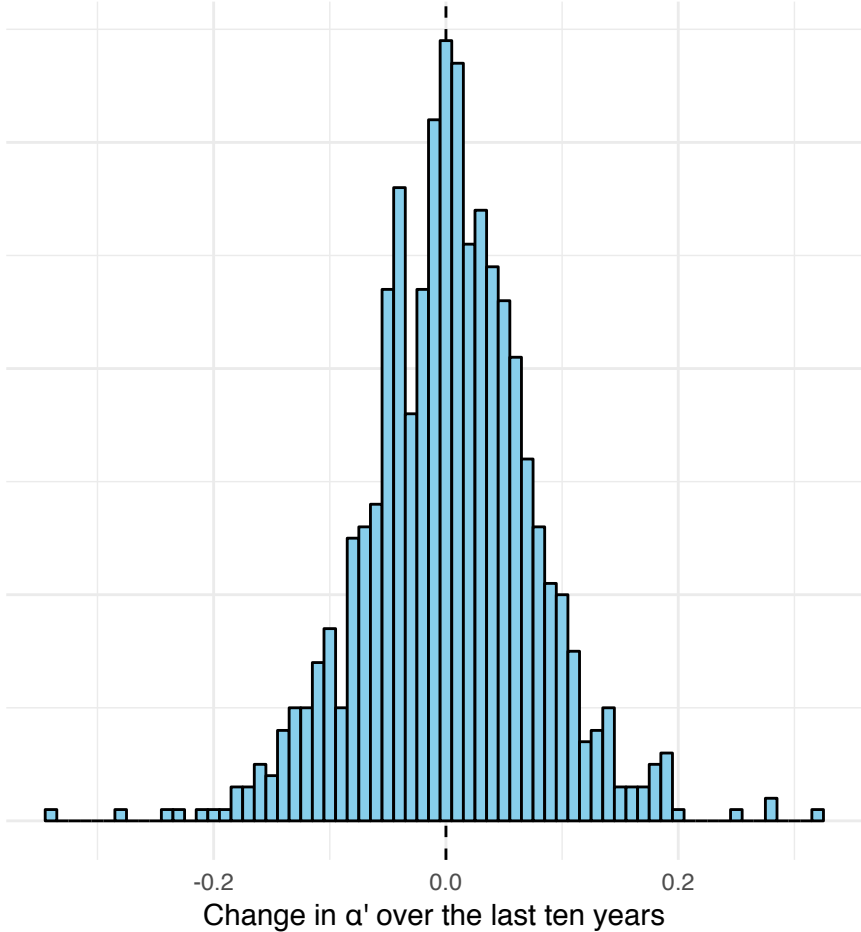

Supplement: S2 Fig — The mean is slightly positive (i.e. 0.004), indicating a mild increase in average α′ with time. (PDF) [file pone.0216128.s002.pdf]

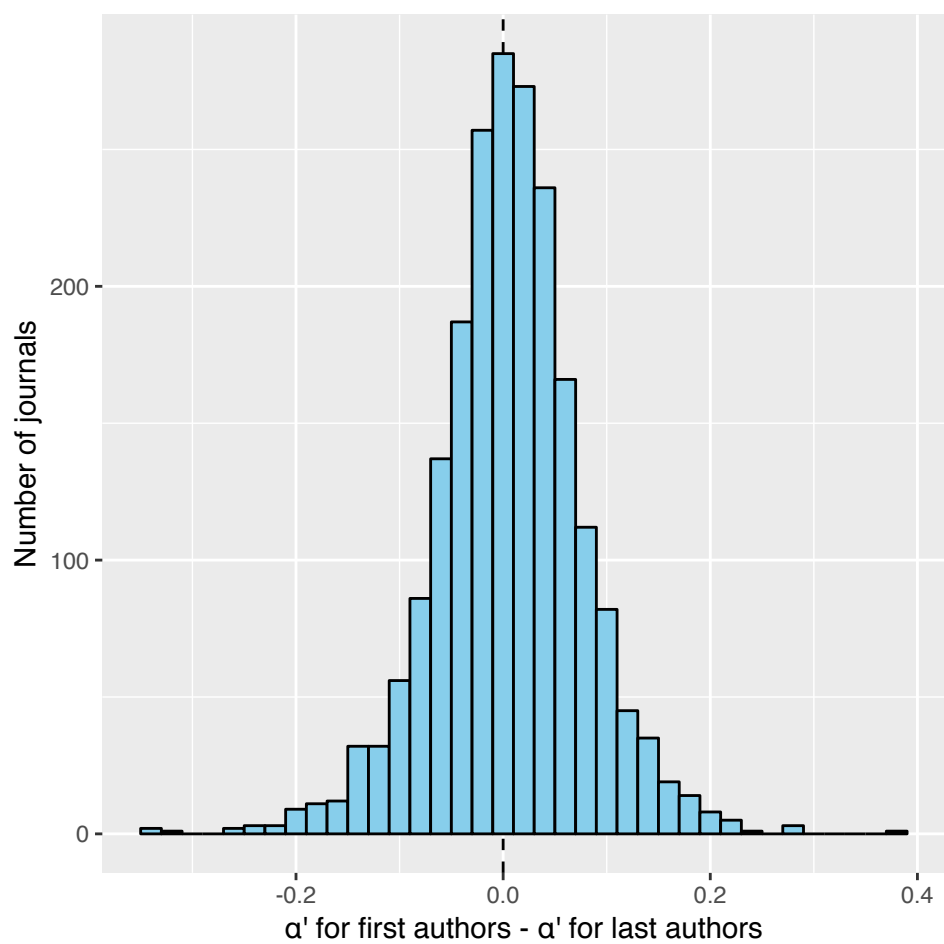

Supplement: S3 Fig — Positive values mean that α′ was higher when calculated for first authors, and negative values mean α′ was higher when calculated for last authors. The mean is very slightly higher than zero, indicating that α′ tends to be higher for first authors. (PDF) [file pone.0216128.s003.pdf]

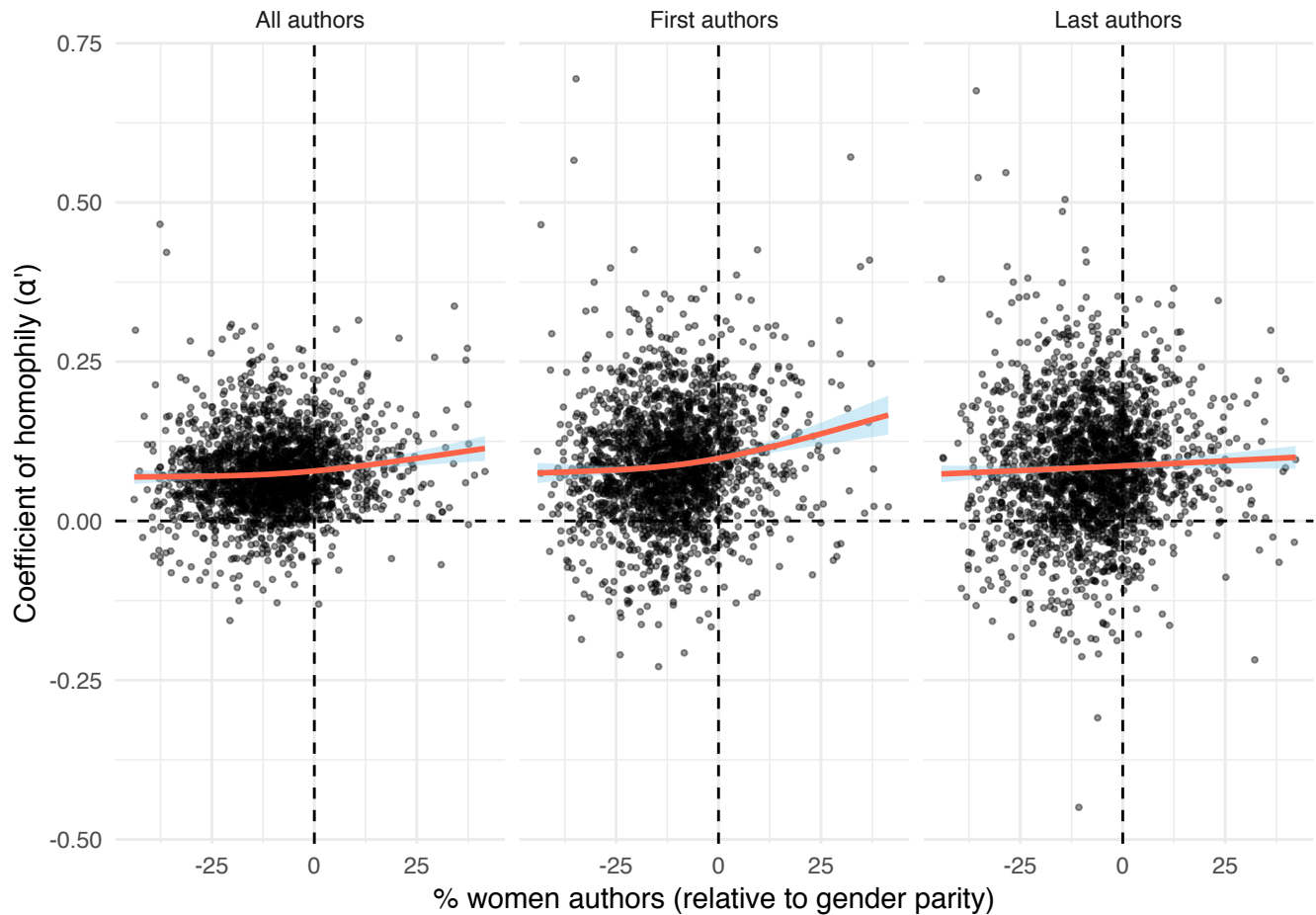

Supplement: S4 Fig — Specifically, journals with 50% women authors or higher tended to have more same-sex coauthorships than did journals in which most authors are men. This relationship held whether α′ was calculated for all authors or first authors only, but not for last authors only. A negative value on the x-axis denotes an excess of men authors, a positive value denotes an excess of women authors, and zero denotes gender parity (i.e. equal numbers of male and female authors). The lines were fitted using generalised additive models with the smoothing parameter k set to 3. (PDF) [file pone.0216128.s004.pdf]

Significant 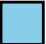 No 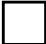 Yes

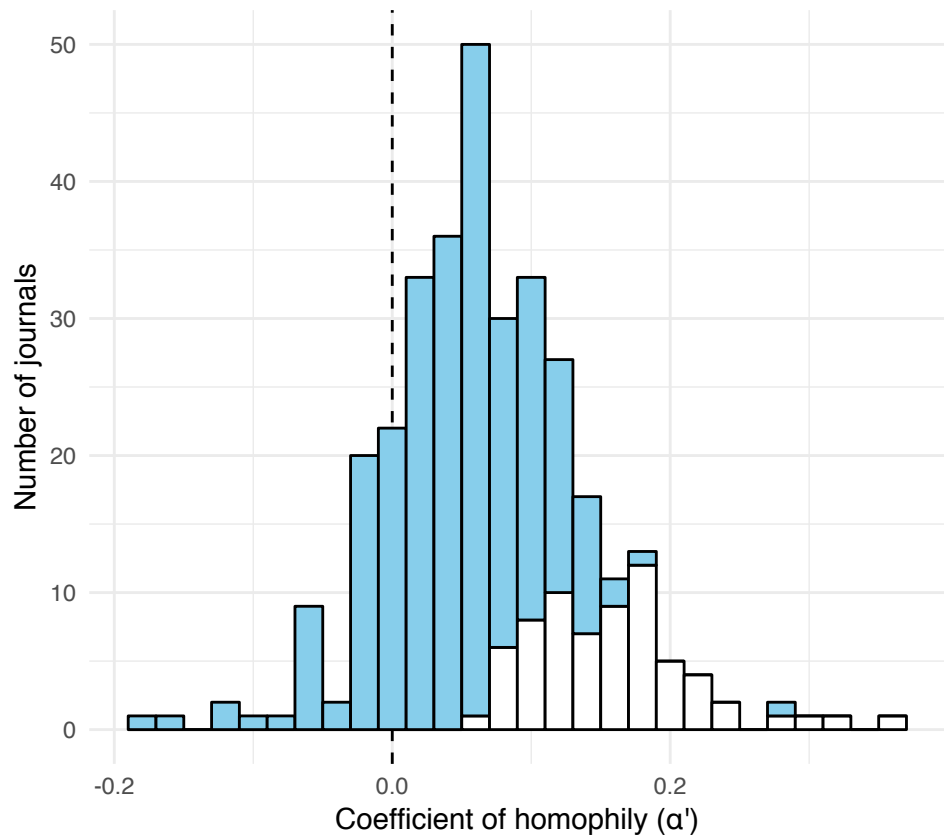

Supplement: S5 Fig — The white areas denote combinations for which α′ differs significantly from zero (p < 0.05, following false discovery rate correction). (PDF) [file pone.0216128.s005.pdf]

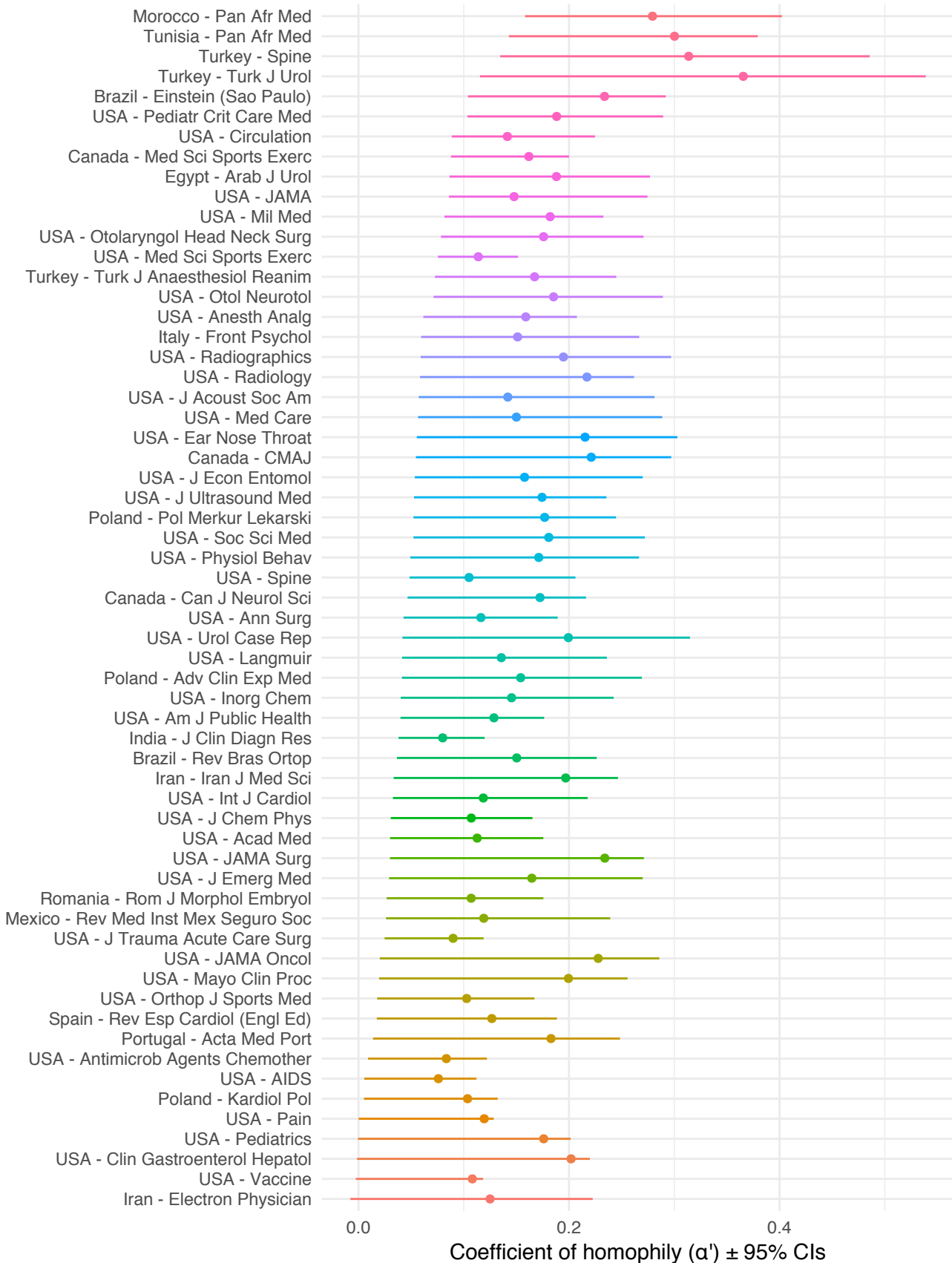

Supplement: S6 Fig — (PDF) [file pone.0216128.s006.pdf]

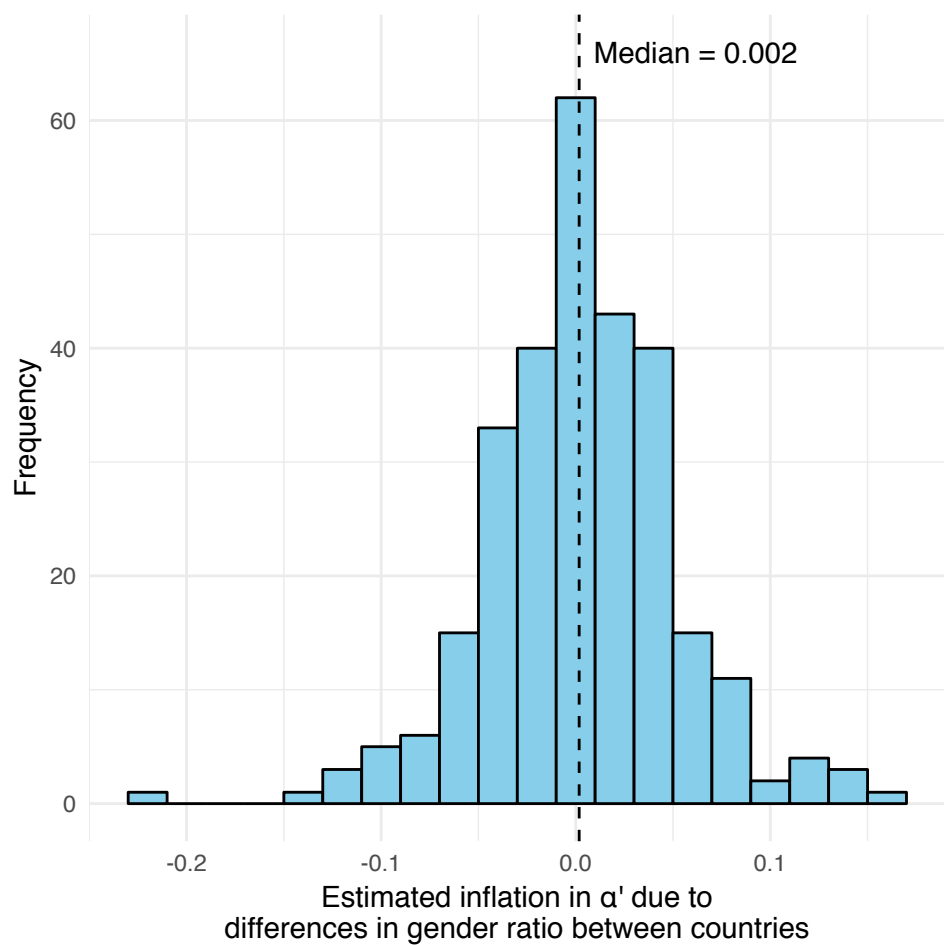

Supplement: S7 Fig — The average inflation in α′ is negligible, suggesting that Wahlund effects resulting from inter-country differences have a negligible effect on our estimates of gender homophily. (PDF) [file pone.0216128.s007.pdf]

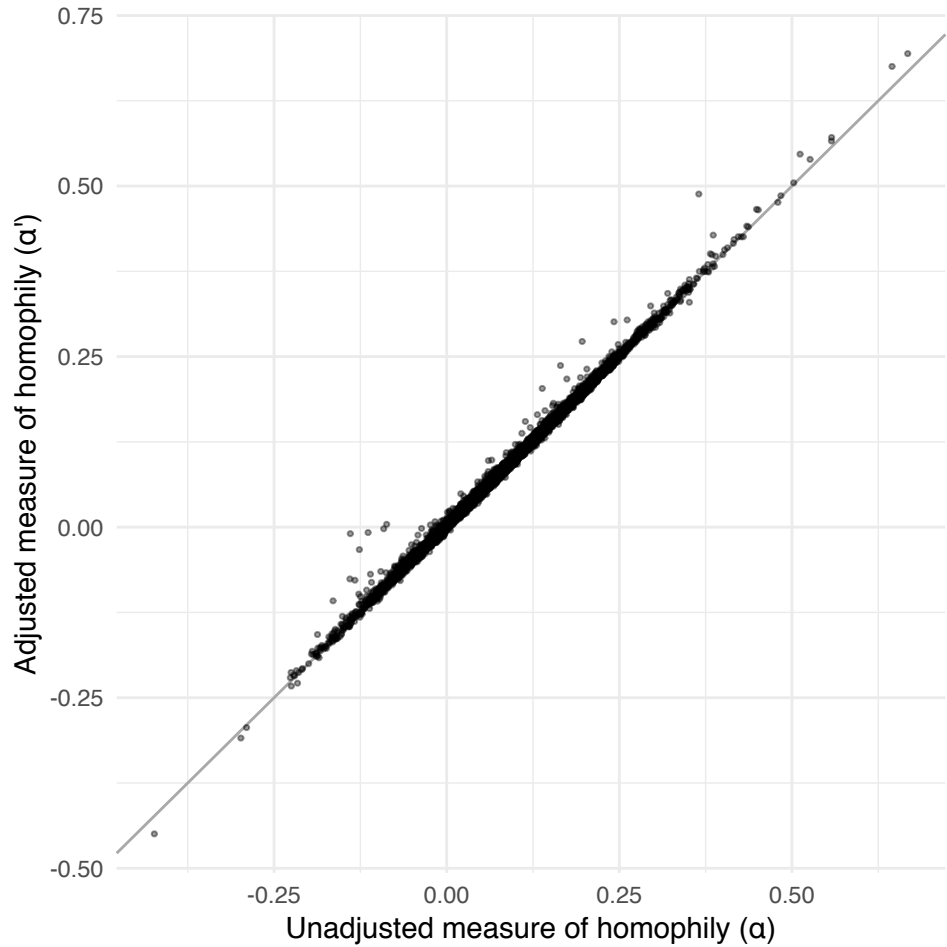

Supplement: S8 Fig — The deviation between α and α′ is greatest for journals for which there is a small sample size (see S9 Fig). (PDF) [file pone.0216128.s008.pdf]

All authors

First authors

Last authors

 $\alpha' - \alpha$ 

0.10

0.05

0.00

2.0 2.5 3.0 3.5 4.0

Log10 sample size (number of papers)

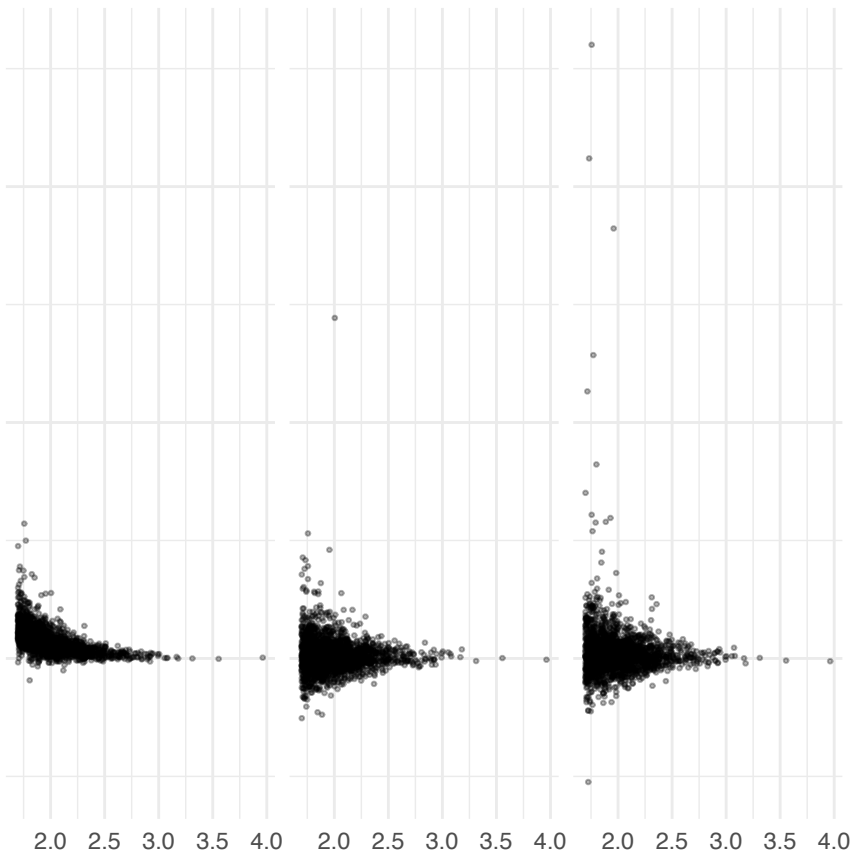

Supplement: S9 Fig — This fits our expectations: because researchers cannot be their own co-authors, small datasets will tend to produce negative estimates of α even if authors assort randomly with respect to gender (see main text). This suggests that α′ is a better measure of homophily and heterophily, though the improvement is trivial in large enough samples. (PDF) [file pone.0216128.s009.pdf]
