# Supplementary material for: Researchers collaborate with same-gendered colleagues more often than expected across the life sciences
Source: PLoS One. 2019 Apr 26;14(4):e0216128. doi: 10.1371/journal.pone.0216128 (PMC6485756; doi:10.1371/journal.pone.0216128)
Supplement: S1 Table — (PDF) [file pone.0216128.s010.pdf]

**Quantity**	**2005 - 2006**	**2015 - 2016**
Number of disciplines	101	107
Number of journals	1192	2116
Number of papers	151652	276879
Number of authors	647634	1311213
Median number of papers per journal	87	87
Median number of authors per journal	371	413
Median number of authors per paper	4	4
