# Supplementary material for: Researchers collaborate with same-gendered colleagues more often than expected across the life sciences
Source: PLoS One. 2019 Apr 26;14(4):e0216128. doi: 10.1371/journal.pone.0216128 (PMC6485756; doi:10.1371/journal.pone.0216128)
Supplement: S2 Table — The significance threshold was p < 0.05, and p-values were adjusted using Benjamini-Hochberg false discovery rate correction. Note that the power of our test is lower for the 2005-2006 data because fewer papers were recovered per journal: thus, it is not meaningful to compare the % significant journals (i.e. 11% vs 24%) between the two time periods. (PDF) [file pone.0216128.s011.pdf]

Year range	Significant heterophily	Significant homophily	Total n journals
2015-2016	2	1472	2116
2005-2006	1	391	1192
